# Poisoning Associated with Consumption of a Homemade Medicinal Liquor — Chongqing, China, 2018

**DOI:** 10.15585/mmwr.mm7116a2

**Published:** 2022-04-22

**Authors:** Chunbei Zhou, Shuquan Luo, Jiang Tang, Linda Quick, Huihui Liu, Yinan Zhao

**Affiliations:** ^1^Chongqing Center for Disease Control and Prevention, Chongqing, China; ^2^Western Chinese Field Epidemiology Training Program, Beijing, China; ^3^Bishan District Center for Disease Control and Prevention, Chongqing, China; ^4^Division of Global Health Protection, Center for Global Health, CDC; ^5^Chinese Field Epidemiology Training Program, CDC China, Beijing, China.

On May 3, 2018, Chongqing Center for Disease Control and Prevention (CQCDC) received a report of 15 persons with numbness of the tongue or limbs and vomiting of unknown etiology; all ill persons had attended an adult birthday luncheon in Bishan District, Chongqing municipality, in southwest China. Initial reports indicated that one person had died. Within 2 hours, CQCDC and Western Chinese Field Epidemiology Training Program staff members launched an investigation that included identification of cases, laboratory testing of drinks, and patient interviews to identify the cause of what appeared to be a poisoning. Among the 15 cases, five persons died. The investigation of this apparent mass intoxication implicated a homemade alcoholic beverage produced from a highly toxic flowering plant in the genus *Aconitum* used in traditional Chinese medicine. Although the risk of aconite toxicity is known, approximately 5,000 cases of aconite poisoning incidents were reported in China, Germany, Japan, and other countries during 1993–2005; most cases of fatal poisoning occurred in China (*1*). This event highlights the importance of enforcing and complying with existing regulations regarding sale and purchase of *Aconitum* species (also known as wolfbane), and of dissemination of critical public health messages. 

## Investigation and Findings

Fifty-three persons had attended the birthday luncheon at a hotel restaurant. A case was defined as the onset of numbness of the tongue or limbs, vomiting, heart palpitations, or sudden death in any person who participated in the birthday luncheon. Demographic information and a list of food and beverages consumed by all attendees were obtained through in-person interviews using a structured questionnaire. Medical records for inpatients were requested and abstracted from hospital and outpatient providers.

A total of 15 cases were identified among the 53 attendees (attack rate = 28%). One patient died on the way to the hospital. Among the remaining 14 patients, all were hospitalized, including nine who were admitted to an intensive care unit; four hospitalized patients died. All patients were men with a median age of 52 years (range = 44–65 years). Among the 15 patients, all reported numbness of the tongue or extremities, 14 reported vomiting, nine reported heart palpitations, and nine reported dizziness (Table). Among the 14 hospitalized patients, 11 had cardiac involvement, including ventricular tachycardia (six patients) and premature ventricular contractions (five patients). Myoglobin was elevated in eight patients, and α-hydroxybutyrate dehydrogenase, a marker of myocardial damage, was elevated in five patients. Other indicators of myocardial damage included abnormal levels of creatine kinase isoenzyme and phosphocreatine kinase. The four patients who died in the hospital all had cardiogenic shock and severe lactic acidosis; two also experienced gastrointestinal bleeding.

**TABLE:** Demographic characteristics, clinical signs and symptoms, laboratory values indicative of myocardial damage, and outcomes of patients with aconite poisoning from homemade medicinal liquor — Bishan District, Chongqing, China, 2018

Patient	Age, yrs	Signs and symptoms	Myoglobin (*μ*g/L)*	α-hydroxybutyrate dehydrogenase (U/L)^†^	Creatine kinase isoenzyme (IU/L)^§^	Phosphocreatine kinase (IU/L)^¶^	Hospitalized	Outcome
A	45	Numbness, vomiting, heart palpitations	NA	NA	NA	NA	No	Died
B**	49	Numbness, vomiting, heart palpitations	81	184	NA	NA	Yes	Died
C**	51	Numbness, vomiting, heart palpitations	247	149	18	100	Yes	Died
D**	55	Numbness, vomiting, heart palpitations	221	239	42	245	Yes	Died
E**	52	Numbness, vomiting	192	216	43	139	Yes	Died
F**	55	Numbness, vomiting, heart palpitations, dizziness	90	NA	NA	NA	Yes	Survived
G	49	Numbness, vomiting, dizziness	41	131	9	129	Yes	Survived
H	65	Numbness, vomiting, heart palpitations, dizziness	NA	198	12	138	Yes	Survived
I	53	Numbness, vomiting, dizziness	58	151	11	122	Yes	Survived
J**	44	Numbness, vomiting, dizziness	98	163	12	220	Yes	Survived
K**	53	Numbness, vomiting, heart palpitations, dizziness	118	NA	NA	NA	Yes	Survived
L**	53	Numbness, vomiting, heart palpitations, dizziness	137	180	39	160	Yes	Survived
M**	52	Numbness, vomiting, heart palpitations, dizziness	NA	139	25	70	Yes	Survived
N	47	Numbness, vomiting	NA	165	24	161	Yes	Survived
O	49	Numbness, dizziness	NA	214	14	148	Yes	Survived

**Abbreviations:** IU = international units; NA = not available; U = units.

* Normal: <72 *μ*g/L.

^†^ Normal: <182 U/L.

^§^ Normal: <25 IU/L.

^¶^ Normal: <200 IU/L.

** Admitted to an intensive care unit.

On the day of the luncheon, the hotel entertained no other guests; all the attendees had eaten breakfast at home. The luncheon started at 12:10 p.m. The 53 attendees sat around five round tables and were all served identical dishes. Beverages consisted of beer, orange juice, soy milk purchased from a local store, and “baijiu,” a Chinese liquor generally distilled from fermented sorghum or other whole grains, provided by the hotel. Some attendees also drank a homemade medicinal liquor prepared by the host. All the drinks except for the homemade medicinal liquor were sealed before being opened and consumed. The hotel tap water was from the local water plant.

**FIGURE 1:**
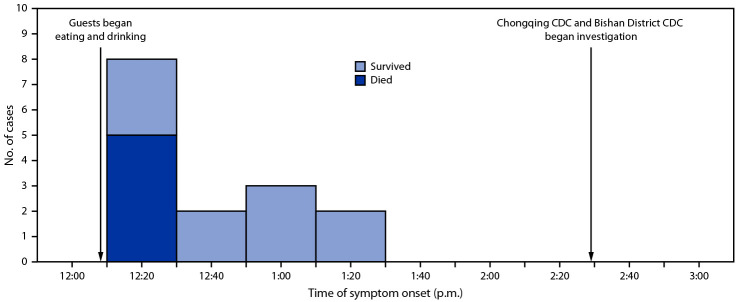
Time line of symptom onset and outcomes among patients with illness and sudden death associated with aconite poisoning from homemade medicinal liquor (N = 15) — Bishan District, Chongqing, China, May 3, 2018 **Abbreviation:** CDC = Center for Disease Control and Prevention.

Symptom onset in the first case was reported at 12:20 p.m., and the last symptom onset occurred 70 minutes later at 1:30 p.m. (Figure 1). The first death occurred at 2:10 p.m. and the last at 10:10 p.m. The median interval from the beginning of the luncheon until symptom onset was 18 minutes among the five fatal cases and 40 minutes among the 10 surviving persons.

**FIGURE 2:**
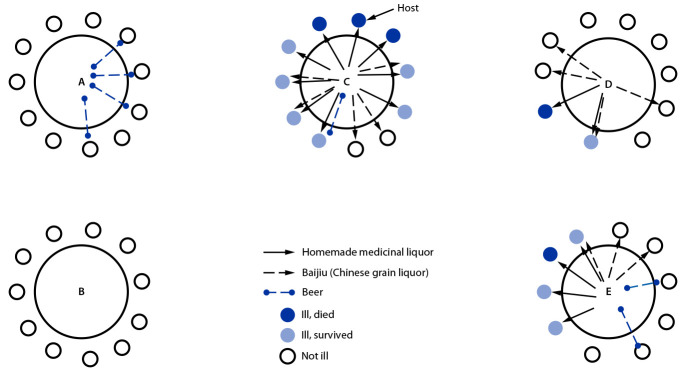
Seat locations of 53 lunch attendees, drinks consumed, and outcomes among patients with illness and sudden death associated with aconite poisoning from homemade medicinal liquor — Bishan District, Chongqing, China, May 3, 2018

The 15 cases occurred among persons seated at three tables (tables C [nine cases among 10 guests and the host], D [two of 10], and E [four of 10]); no cases occurred among the 11 guests seated at table A or the 11 at table B (Figure 2). All 15 attendees who became ill drank the medicinal liquor (100%), including nine who drank only the medicinal liquor (five also consumed the baijiu, and one also drank beer). Five of the nine patients who drank only the medicinal liquor died. All attendees who drank the medicinal liquor became ill. None of the 10 hotel employees serving became ill, nor were any similar cases reported in the surrounding area.

The host of the luncheon had made the liquor, and also died after drinking it; therefore, details of how it was prepared were not available. His wife recalled that aconite roots were given to her husband by a friend several years earlier. The medicinal liquor was prepared several years before, distilled spirits were added to the roots for soaking, and the mixture was kept at the host’s home until the day of the birthday luncheon. The host’s wife speculated that aconite roots were mistaken by her husband for *Lepidium meyenii*, a nontoxic plant root also reported to be used as medicine.

Examination of the plant roots remaining in the bottle of medicinal liquor were morphologically similar to aconite roots used in traditional Chinese medicine. Samples were sent to the CQCDC Physicochemical and Toxicology Testing Laboratory. The alkaloid database was built by Thermo Trace Finder software (https://www.thermofisher.com/us/en/home.html), and rapidly screened for 42 alkaloids by ultra-performance liquid chromatography-quadrupole/electrostatic field orbitrap high-resolution mass spectrometry. Bullatine A, bullatine B, aconitine, and mesaconitine, which are aconite byproducts, were detected in the medicinal liquor, and all four of these *Aconitum* alkaloids are known to be toxic. Bullatine A and aconitine concentrations were 81.9 *μ*g/mL (a highly toxic dose) and 0.393 *μ*g/mL, respectively. The concentrations of the other two components were extremely low. On the basis of the evidence, the medicinal liquor was suspected to be the cause of the poisoning, most likely the result of bullatine A ingestion. No toxicology testing was performed on any of the persons who were poisoned.

## Public Health Response

The local government immediately responded to this public health emergency. The response included patient treatment and joint investigation by public security (police), health departments, and regulatory authorities. At the conclusion of the investigation, public health safety messages were disseminated through the media to ensure public awareness of the poisonous qualities of *Aconitum *species.

## Discussion

This investigation provides compelling evidence that this mass intoxication was caused by homemade medicinal liquor containing *Aconitum *alkaloids. *Aconitum lycoctonum* is included in the *Aconitum* genus of >250 species of flowering plants belonging to the Ranunculaceae family. These herbaceous perennial plants are native to the mountainous parts of the Northern Hemisphere, growing in the moisture-retentive but well-draining soils of mountain meadows, mainly in southwestern China. Among medicinal herbs, *Aconitum* species are unique: while reportedly considered a beneficial herb root used historically and extensively for medicinal purposes in China, *Aconitum* species, also known as wolfbane, are highly toxic, and if not prepared correctly, consumption can be rapidly fatal.

Aconite tubers and roots are traditionally used in medications to treat pain and a range of diverse ailments, including diarrhea, edema, asthma, various tumors, rheumatism, rheumatoid arthritis, and other inflammatory disorders (*2*,*3*). They are frequently used in traditional Chinese medicine, and, although the sale of unprocessed *Aconitum* species is prohibited, they can be illicitly acquired in open markets in China, especially in Yunnan and Guizhou provinces. These sales of unprocessed roots occur, even though the activity is forbidden by drug safety regulation stipulated by the State Administration for Market Regulation, and even though the pharmaceutical use of drugs containing *Aconitum* alkaloids is strictly monitored in China.

The medicinal use of *Aconitum* species requires strict adherence to processing and cooking guidance, which is based on the prescribed dosage (*4*). Only licensed manufacturers or authorized practitioners are allowed to prepare *Aconitum* species for medical use. Traditional Chinese methods for processing herbs (páo zhì), including heating, soaking, and boiling for several hours, are reported to enhance the properties or reduce or eliminate toxicity, and in the case of *Aconitum *species, convert the alkaloids to comparatively less toxic or nontoxic derivatives (*5*), so that medicinal liquors containing aconite roots may be safely drunk for health promotion (*3*). If the products are misused or not prepared correctly, aconite ingestion can result in rapid death. Aconite roots and leaves, as well as honey made from aconite nectar*,* are all highly toxic. If they are not properly decocted for oral consumption, *Aconitum* plant products can be safely used only when applied topically. As little as 0.2 mg (200 *μ*g) of *Aconitum* alkaloids can cause poisoning, and 2.0 mg (2,000 *μ*g) is sufficient to result in death.

Although the risk of aconite toxicity is known, in some areas of China, *Aconitum* plant products are still sought for their purported health benefits. Approximately 5,000 cases of aconite poisoning were reported in China, Germany, Japan, and other countries during 1993–2005; most cases of fatal poisoning occurred in China (*1*). Aconite roots contain various chemical constituents, such as aconitine, mesaconitine, and bullatine A, which have significant pharmacologic activity and are also toxic. The *Aconitum* alkaloids primarily affect the central nervous system, heart, and muscle cells (*6*–*8*), with cardiac damage the most serious consequence. As was the case with most of the victims of this intoxication, severe cases of cardiac toxicity from consumption of aconitine-containing herbal preparations typically manifest clinically as ventricular tachycardia and fibrillation, frequently resulting in death. *Aconitum* alkaloids bind to cardiac muscle cell receptors that regulate sodium-ion channels, preventing repolarization of cells, and resulting in paralysis and death (*9*,*10*).

The findings in this report are subject to at least three limitations. First, no toxicology testing was done on the victims. However, the hypothesis that the toxicant was a homemade medicinal liquor containing products from *Aconitum* species is supported by the fact that 100% of persons who drank it and none of those who did not drink it became ill. Second, it was not possible to ascertain the precise volume of medicinal liquor consumed by each person, but the amount consumed by each person ranged from 5–125 mL (approximately 1–25 tsp). On the basis of the concentration of residue identified by toxicology testing (81.9 *μ*g/mL), it can be inferred that as little as 25 mL (approximately 5 tsp) contained sufficient *Aconitum* alkaloids to be lethal. Finally, the source of *Aconitum* species could not be ascertained. In a retrospective analysis of case reports of aconite poisoning in China during 2004–2015, the most commonly reported route of exposure was drinking medicinal liquors containing *Aconitum* alkaloids; concurrent ethanol ingestion could rapidly increase the absorption of *Aconitum* alkaloids into the blood (*3*), which might have contributed to the rapid onset and five fatal cases in persons who drank only the medicinal liquor in this event. In addition, the absence of labeling of the root might have contributed to its mistaken inclusion in the recipe.

Although *Aconitum* species can be used medicinally if properly prepared by a licensed manufacturer or authorized practitioner, ingestion can also result in fatal intoxication; therefore, regulations and public health messaging to increase awareness of the toxicity of *Aconitum *species can help prevent inadvertent aconite poisoning. Emphasizing the danger of drinking medicinal liquors containing *Aconitum* alkaloids and the identification of plants that are easily confused are critical to ensure the safe use of *Aconitum *species. Conspicuous labeling of bottles containing homemade brews might prevent misuse, especially oral consumption of products that are only appropriate for topical use. In addition, existing safety monitoring of materials containing *Aconitum* alkaloids and surveillance of the poison control network are critical.

**Summary**

**What is already known about this topic?**

Aconite tubers and roots are used to prepare traditional Chinese medicinal drinks; fatal aconite poisonings resulting from improper production occur every year.

**What is added by this report?**

Fifteen cases of poisoning, including five deaths, were reported among persons who attended a birthday luncheon at a Chongqing hotel. Intoxication was most likely caused by homemade unlabeled medicinal liquor containing extremely high concentrations of toxic *Aconitum* alkaloids.

**What are the implications for public health practice?**

Public prevention strategies should focus on increasing recognition of the toxicity of *Aconitum* species and the identification of similar plants, strengthening existing regulation of the sale of raw materials containing *Aconitum* alkaloids, and appropriately labeling homemade products.
